# 864. Pulmonary Function Evaluation Following Veno-venous Extracorporeal Membrane Oxygenation (VV-ECMO) Support for Severe Smoke Inhalation Injury

**DOI:** 10.1093/jbcr/irag033.334

**Published:** 2026-04-06

**Authors:** Michelle K Hughes, Anne Seyferth, Annie Cate Schmidt, William Hughes

**Affiliations:** Jefferson Burn Center, Thomas Jefferson University Hospital, Philadelphia, PA; Sidney Kimmel Medical College, Philadelphia, PA; Jefferson Burn Center, Thomas Jefferson University Hospital, Philadelphia, PA; Jefferson Burn Center, Thomas Jefferson University Hospital, Philadelphia, PA

## Abstract

**Introduction:**

Although pulmonary complications are a major cause of morbidity and mortality in burn patients, little is known about lung function after inhalation injuries causing respiratory failure. Descriptions of pulmonary function testing (PFTs) following severe inhalation injuries have previously been published; to our knowledge, this is the first report of PFT data obtained from patients with severe inhalation injuries requiring VV-ECMO support.

**Methods:**

Two patients with flame and inhalation injuries were studied. Neither had a history of lung disease or subjective functional limitation. Both patients developed respiratory failure refractory to maximal conventional management. They were placed on ECMO and underwent support for extended courses of greater than three weeks. The patients were weaned from ECMO and mechanical ventilation and ultimately underwent tracheostomy decannulation. Pulmonary function tests were performed just prior to discharge.

**Results:**

In the post-COVID era, our institution has successfully utilized ECMO support in three patients with severe respiratory failure from smoke inhalation injuries. One patient received just two days of ECMO and was excluded, as the impact was considered minimal. Patient 1 had severe restrictive disease based on a forced vital capacity (FVC) of 1.57 L (41 % of predicted using NHANES III reference data); we were unable to collect diffusing capacity testing. Patient 2 had moderate restrictive disease based on a forced expiratory volume in one second (FEV1) of 2.12 L (62% of predicted), an FVC of 2.48 L (60% of predicted), a normal FEV1/FVC ratio, and a total lung capacity (TLC) of 3.27 L (54% of predicted). Patient 2 also had a severe reduction in diffusing capacity, with a diffusing capacity for carbon monoxide (DLCO) (corrected for a hemoglobin of 9.5 g/dl) of 12.23 ml/min/mmHg (44% of predicted). Follow up PFTs will be performed to evaluate for permanent damage or improvement.

**Conclusions:**

Pulmonary injury is the leading cause of mortality and morbidity in burn patients. For cases refractory to conventional support, ECMO putatively brings distinct advantages and risks; however, these are difficult to measure. ECMO has been used to facilitate operative debridement, early mobilization, and reduced stress from mechanical ventilation; however, it also imposes significant hematological and immunological dangers, some of which may be more substantial for patients with burn injuries. Furthermore, increased costs and limited availability make the practical use of ECMO challenging. Data, including PFTs, may be a helpful comparator in future analyses of the impact of ECMO on inhalation injury survivors.

**Applicability of Research to Practice:**

This study demonstrates PFT outcomes data for patients with severe smoke inhalation injuries requiring VV-ECMO support. Future analyses using such data may guide rehabilitation strategies and inform clinical decision-making.

**Funding for the study:**

N/A.

